# Decreased circulating Zinc levels in Parkinson’s disease: a meta-analysis study

**DOI:** 10.1038/s41598-017-04252-0

**Published:** 2017-06-20

**Authors:** Ke Du, Ming-Yan Liu, Xin Zhong, Min-Jie Wei

**Affiliations:** 1 School of Pharmacy, Department of Pharmacology, China Medical University, Shenyang, Liaoning 110122 China; 2 Liaoning Key Laboratory of molecular targeted anti-tumor drug development and evaluation, Shenyang, Liaoning 110122 China

## Abstract

There is no consensus on the involvement of zinc (Zn) dysfunctions in Parkinson’s Disease (PD). We performed a meta-analysis to evaluate whether circulating Zn levels in the serum, plasma, and cerebrospinal fluid (CSF) are altered in PD. Twenty-three published studies were selected by searching the databases of PubMed and China National Knowledge Infrastructure (CNKI). A total of 803 PD patients and 796 controls, 342 PD patients and 392 controls, and 135 PD patients and 93 controls were included to study Zn levels in the serum, plasma, and CSF, respectively. Our meta-analysis showed that the serum Zn levels were significantly lower in PD patients compared with health controls (SMD = −0.59; 95% CI [−1.06, −0.12]; P = 0.014). A reduced Zn levels in PD patients were found when serum and plasma studies were analyzed together (SMD = −0.60, 95% CI [−0.98; −0.22]; p = 0.002). PD patients had a tendency toward reduced CSF Zn levels compared with health controls (SMD = −0.50; 95% CI [−1.76, 0.76]; P = 0.439), but no statistical significance was obtained and this data did not allow conclusions due to a small sample size of CSF studies. This study suggests that reduced Zn levels in the serum and plasma are associated with an increased risk for PD.

## Introduction

Parkinson disease (PD) is a progressive neurodegenerative disorder, and the incidence of the disease rises steeply with age. The lifetime risk of developing the disease is approximately 1.5%^1, 2^. PD is most commonly associated with motor symptoms, including tremor, rigidity, slowness of movement, and postural instability. The pathological changes of the disease are characterized by a significant loss of dopaminergic neurons in the substantia nigra pars compacta and the presence of intraneuronal proteinaceous cytoplasmic inclusions termed Lewy bodies^3–5^. Although the etiology of PD is largely unknown, PD is associated with many etiological factors including ageing, genetic susceptibility, and disturbances in trace element homeostasis^6–9^.

Zn is an essential element for human health, and plays crucial and multiple roles in central nervous system (CNS). Zn are rich in the hippocampus and cerebral cortex^10, 11^. Zn deprivation influences Zn homeostasis in the brain, leading to changes in behavior, learning, memory, and emotional stability^12^. Disturbance of Zn homeostasis has been found to be associated with the pathogenesis of many neurodegenerative disorders in CNS, such as PD, Alzheimer’s disease and amyotrophic lateral sclerosis^13–18^. Recently, it has been found that Zn ions directly binds to the peptide fragments from the Parkinson’s disease gene Park9^19–21^. Removal of Zn from Park2 causes nearly complete unfolding of the protein and loss of its activity^22^. However, ambiguous and contradictory results exist in the literature regarding circulating Zn levels in PD patients. For example, some studies reported a decrease of circulating Zn in patients with PD compared with health controls^23–33^, and other studies found no significant difference or even increased Zn levels in PD patients^34–45^.

To evaluate whether Zn levels is altered in PD, we performed a meta-analysis to compare the zinc levels in the serum, plasma, and CSF in PD patients versus health controls. In this article, we applied this statistical method combining the results of different studies on the levels of zinc in PD, which strengthens the power of the conclusions.

## Methods

### Search strategy and study selection

The databases of PubMed and China National Knowledge Infrastructure (CNKI) were searched for published studies from inception to 2016 reporting the relationships between circulating zinc levels and PD. We entered the keywords “Parkinson’s disease”, “zinc”, “serum”, “plasma”, “CSF”, “metals” and their combinations in English or Chinese language. No language limits were applied. Eligible articles were retrieved from the above data. In addition, we reviewed references of relevant articles. Eligible studies were selected according to the following inclusion criteria: (1) human study, (2) case-control study, and (3) sample size and Zn levels in serum, plasma or CSF were provided for both PD subjects and health controls. Exclusion criteria included: (1) repeated or overlapped publications, (2) animal study, (3) review, abstracts, letters, and case reports, and (4) studies not providing Zn levels for health controls.

### Data extraction

Two authors independently (D.K. and Z.X.) assessed each study and extracted the relevant information: the first author last name, year of publication, sample size, mean age of the participants, the percentage of women included in PD and control sample, and the technique used. The mean Zn levels and the corresponding standard deviations were also recorded, or, were estimated from median, range, and the sample size, if they were not directly reported^46^.

### Statistical analysis

We used STATA 12.0 (Stata, College Station, TX, USA) to perform all the statistical analyses. To obtain the pooled estimate of the difference in the Zn level between PD and control group, a random effects model was applied, when significant heterogeneity was observed. Otherwise, the fixed effects mode was adopted. Random-effects models were used to combine study-specific Standardized Mean Difference (SMD), which standardizes the outcome for each individual study to the effect size found in terms of the standard deviation observed in the study. Heterogeneity was evaluated using Chi-square and the I-square test.

To study the causes of heterogeneity, a subgroup analysis was conducted to assess the impact of the method used for measuring Zn levels and the geographic location of studied population. Meta-regression analysis was also performed to analyze the potentially important covariates of patient’s mean age and gender distribution (proportion of women), and to analyze whether the continuous variables had moderating effects on the outcomes of the meta-analysis. Sensitivity analysis was used to investigate the influence of a single study on the overall effect estimate by omitting one study at a time during repeated analyses. Potential publication bias was evaluated by funnel plot with the Egger test. Temporal effect was also evaluated with a cumulative meta-analysis. P-values less than 0.05 were considered statistically significant.

## Results

### Study Selection and characteristics of eligible studies

**Figure 1:**
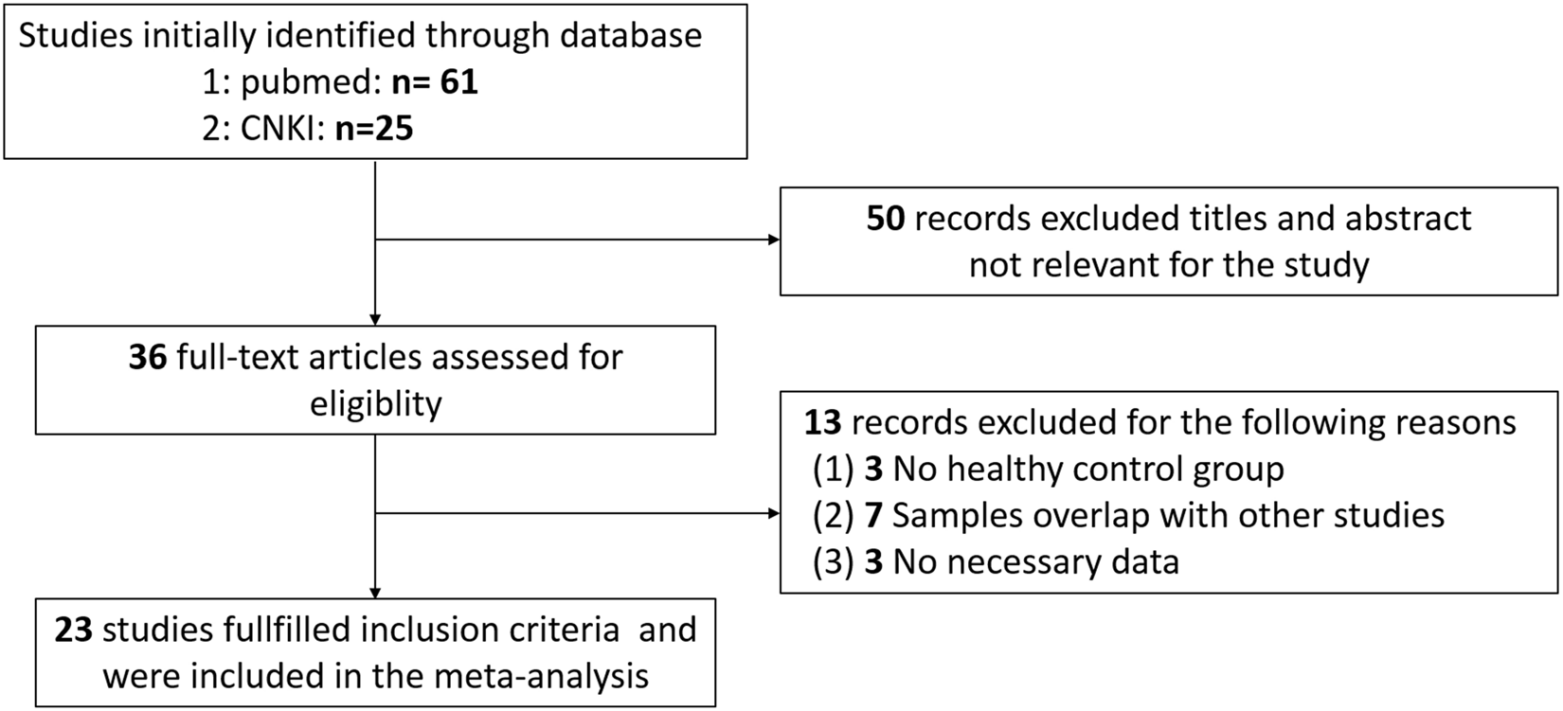
Meta-Analysis flowchart of the literature search.

Thirty-six potential studies were identified after an initial search from the Pubmed, CNKI and the references of relevant articles. After further screening, twenty-three studies were finally selected to be related to our meta-analysis (a total of 1306 cases and 1294 controls). The flow diagram of the search process is shown in Fig. 1. Patient samples ranged from 17 to 238 subjects. The mean age of PD patients ranged from 55.4 to 71.1 years and the percentage of female patients ranged from 8% to 62%. The data of mean age was missing in three of these studies, and the data of percentage of female was missing in two of these studies. The analyzed population was European or American in 8 studies, Asian in 14, and African in 1. The detailed characteristics are presented in Table 1.

**Table 1:** Characteristics of studies on the association between Zn levels and PD risk.

Author	Year	Country	PD Patients				Health Controls				Method
			n	Gender	Age	Zinc concentration:	n	Gender	Age	Zinc concentration:	
				(% Female)	(Mean ± SD)	mean ± SD (µg/l)		(% Female)	(Mean ± SD)	mean ± SD (µg/l)	
*studies on serum*											
Luo	1987	China	30	30	61.5 ± 8.4	812.4 ± 137.3	30	37	>45	909.0 ± 152.8	AAS
Pan	1991	China	62	39	55.4 ± 9.1	67.3 ± 90.2	33	39	52.3 ± 12.8	126.9 ± 168.0	ICP-AES
Jiménez-Jiménez	1998	Spain	37	62	65.7 ± 8.8	820.0 ± 230.0	37	57	62.4 ± 17.8	770.0 ± 170.0	AAS
Yang	2001	China	22	36	—	273.2 ± 171.8	30	40	—	343.8 ± 189.7	ICP-AES
Forte	2004	Italy	26	8	64.9 ± 10.8	669.0 ± 118.0	13	54	63.8 ± 13.7	705.0 ± 91.1	ICP-AES
Hegde	2004	India	52	46	58.2 ± 4.7	491.5 ± 108.7	25	48	55.4 ± 6.4	588.3 ± 65.3	ICP-AES
Qureshi	2006	France	36	36	71.1 ± 15.9	846.7 ± 82.2	21	38	62.0 ± 11.0	890.0 ± 20.0	AAS
Alimonti	2007	Italy	71	25	65.9 ± 9.4	720.0 ± 27.5	124	35	44.8 ± 12.7	795.0 ± 32.3	ICP-AES
Gellein	2008	Norway	33	52	—	994.0 ± 331.0	99	52	—	992.0 ± 304.0	ICP-MS
Nikam	2009	India	40	—	—	757.0 ± 92.0	40	—	—	985.0 ± 82.5	AAS
Squitti	2009	Italy	93	42	70.2 ± 9.0	851.2 ± 144.5	76	43	68.0 ± 8.5	769.4 ± 30.9	5-Br-PAPS
Brewer	2010	America	30	40	67.4 ± 8.2	774.0 ± 94.0	29	69	68.6 ± 6	827.0 ± 139.0	AAS
Ahmed	2010	India	45	38	55.6 ± 3.2	430.0 ± 40.0	42	45	57.6 ± 9.1	590.0 ± 70.0	ICP-MS
Zhang	2010	China	17	59	66.0 ± 9.0	1379.0 ± 402.0	10	60	64.0 ± 4.0	1274.0 ± 183.0	AAS
Fukushima	2011	China	71	42	63.7 ± 9.7	1060.0 ± 460.0	71	42	63.4 ± 9.7	1080.0 ± 480.0	ICP-AES
Zhou	2011	China	40	38	64.6 ± 11.5	752.0 ± 266.0	40	38	63.4 ± 10.7	705.0 ± 239.0	ICP-AES
Youne	2013	Tunisia	48	46	65.8 ± 10.2	627.6 ± 170.0	36	61	59.7 ± 12.0	581.9 ± 202.0	AAS
Hou	2014	China	50	32	66.0 ± 8.0	564.0 ± 133.0	40	33	64.0 ± 12.0	642.0 ± 133.0	ICP-AES
*studies on plasma*											
Fang	1994	China	74	39	56.2 ± 10.8	779.3 ± 474.7	66	33	53.7 ± 14.2	1167.7 ± 489.7	AAS
Kocaturk	2000	Turkey	30	—	64.0 ± 0.0	872.9 ± 130.0	24	—	61.0 ± 0.0	894.6 ± 130.0	AAS
Zhao	2013	China	238	49	66.6 ± 11.3	923.0 ± 338.0	302	49	65.6 ± 12.2	1293.0 ± 385.0	AAS
*studies on CSF*											
Jiménez-Jiménez	1998	Spain	37	62	65.7 ± 8.8	100.0 ± 60.0	37	57	62.4 ± 17.8	170.0 ± 140.0	AAS
Qureshi	2006	France	36	36	71.1 ± 15.9	106.0 ± 18.0	21	38	62.0 ± 11.0	161.0 ± 31.0	AAS
Alimonti	2007	Italy	42	14	64.5 ± 10.7	27.7 ± 9.0	20	15	66.2 ± 14.7	32.3 ± 11.4	ICP-AES
Hozumi	2011	Japan	20	55	68.7 ± 5.8	14.5 ± 7.6	15	60	48.4 ± 22.2	5.3 ± 3.3	ICP-MS

ICP-MS, inductively coupled plasma-mass spectrometry; ICP-AES, inductively coupled plasma-atomic emission spectrometry; AAS, atomic absorption spectrometry; 5-Br-PAPS, 2-(5-bromo-2-pyridylazo)-5-(N-phenyl-N-sulfopropylamino) phenol.

### Meta-analysis of Zn levels in the serum

**Figure 2:**
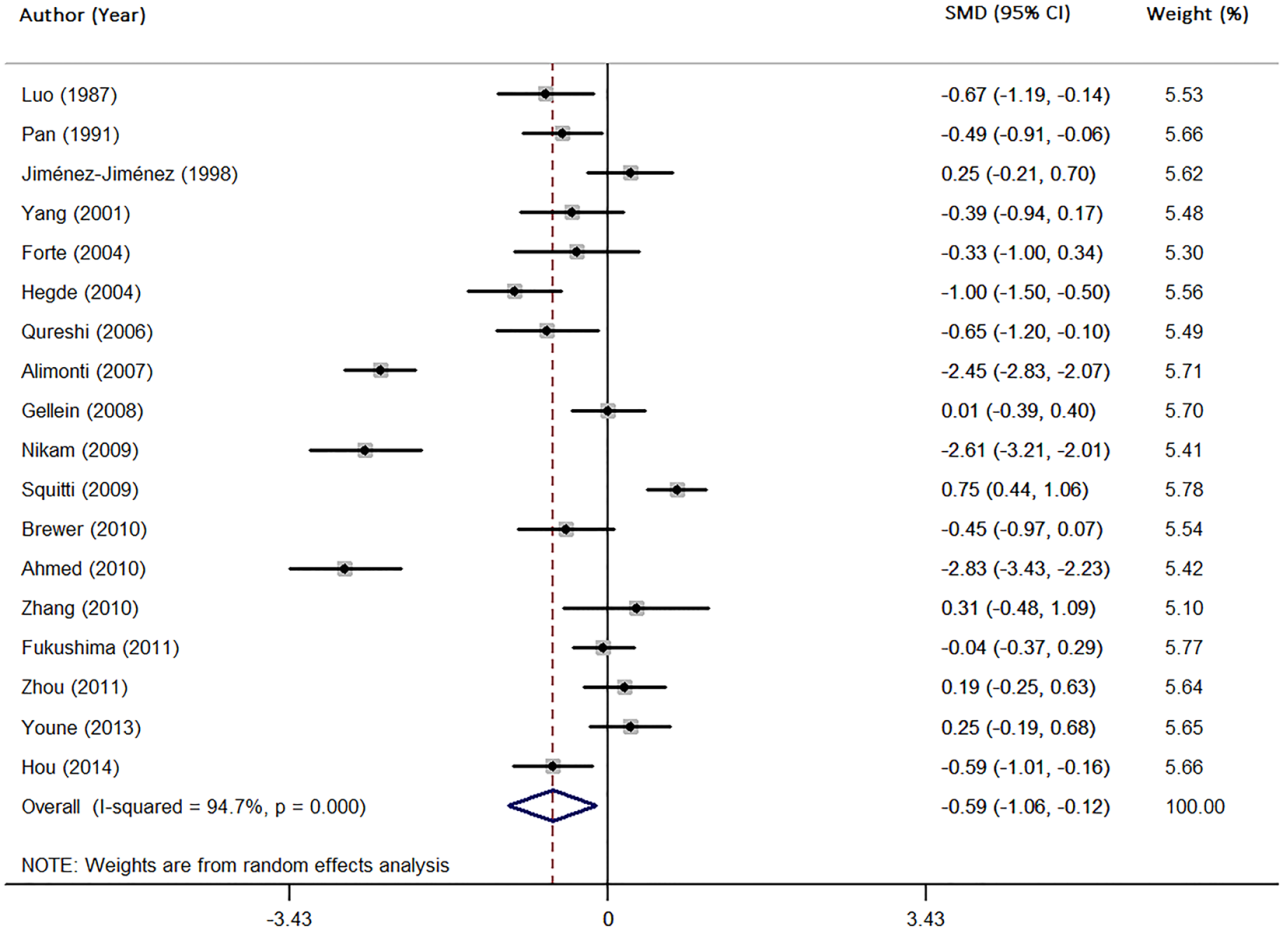
Forest plot for random-effects meta-analysis on differences in serum Zn between PD patients and health controls. 18 studies encompassing a sample of 1599 subjects. The horizontal lines represent 95% CI. The sizes of the shaded squares are proportional to study weight. SMD, Standardized mean difference; CI, confidence interval.

Data from 18 studies was analyzed in a random-effects model to compare the serum Zn levels in PD patients and health controls (Table 1). The pooled sample size consisted of 1599 subjects including 803 PD and 796 controls. PD patients had significantly lower levels of serum Zn levels than health controls (SMD = −0.59; 95% CI [−1.06, −0.12]; P = 0.014) (Fig. 2). Significant heterogeneity was found among these studies (I^2^ = 94.7%, P = 0.000). Subgroup analysis by the method used for measuring Zn levels showed a high heterogeneity in each subgroup. Additionally, the subgroup analysis by geographic locations, showed that the heterogeneity was high in European populations and Asian populations. Neither the method for measuring Zn levels nor the geographic location was a main source of heterogeneity (Table 2). In meta-regression analyses, age and gender had no moderating effects (mean age: P = 0.076; gender: P = 0.121). Sensitivity analyses showed that no single study significantly changed the results. The results of cumulative analysis excluded a temporal effect. Furthermore, Egger’s regression asymmetry test did not suggest publication bias (P = 0.163) in the present meta-analysis.

**Table 2:** Meta-analysis of studies on serum.

Subgroups	n of studies	SMD (95% CI)	I^2^	P-value
All studies	18	−0.59 (−1.06, −0.12)	94.7%	0.000
Methods				
ICP-MS or ICP-AES	10	−0.79 (−1.42, −0.16)	94.9%	0.000
AAS	7	−0.51 (−1.21, 0.20)	91.8%	0.000
other	1	0.75 (0.44, 1.06)	—	—
Geographic locations				
Asia	10	−0.80 (−1.39, −0.22)	93.0%	0.000
Europe or America	7	−0.41 (−1.31, 0.49)	96.6%	0.000
Africa	1	0.25 (−0.19, 0.68)	—	—

SMD with the corresponding 95% CI and

p value, the I^2^ statistic for overall and subgroup analyses. SMD, Standardized mean difference; CI, confidence interval.

### Meta-analysis of Zn levels in the plasma

**Figure 3:**
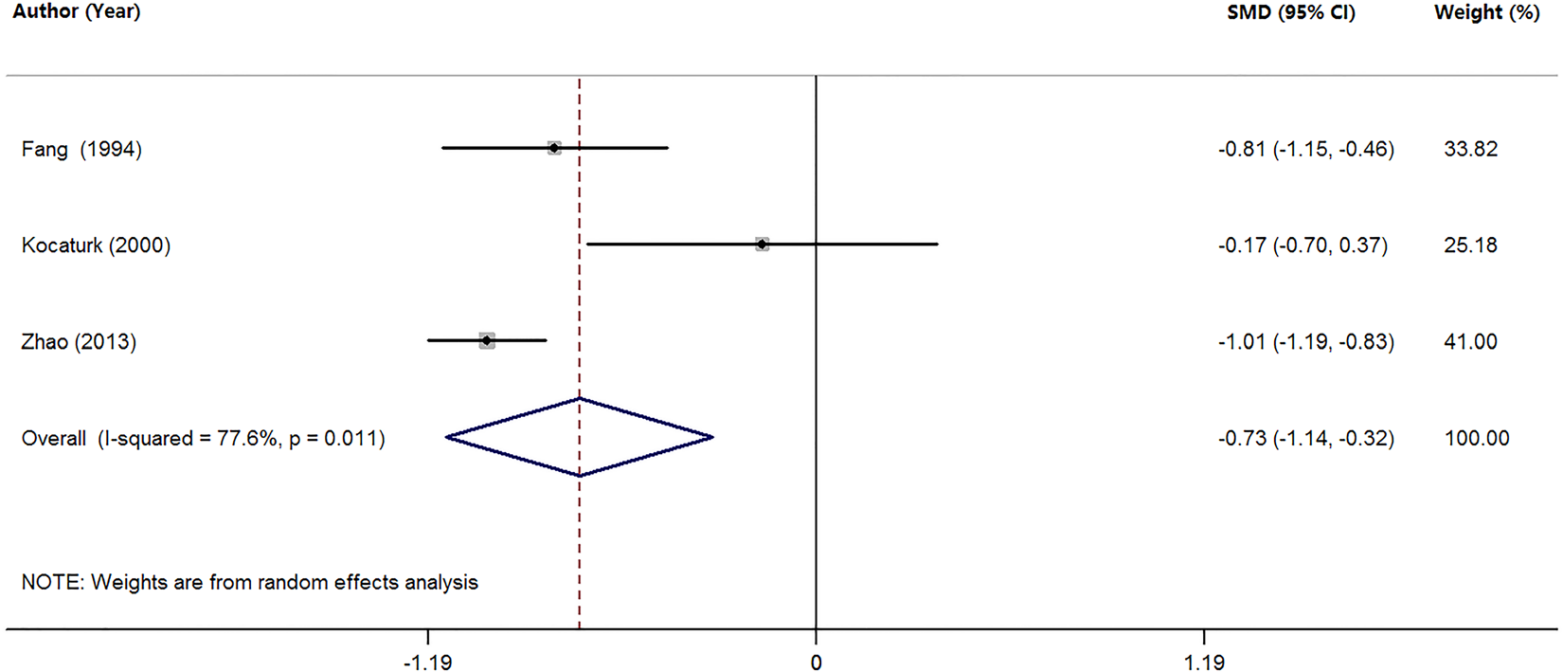
Forest plot for random-effects meta-analysis on differences in plasma Zn between PD patients and health controls. 3 studies encompassing a sample of 734 subjects. The horizontal lines represent 95% CI. The sizes of the shaded squares are proportional to study weight. SMD, Standardized mean difference; CI, confidence interval.

Three studies were selected with a pooled sample size of 734 subjects including 342 PD patients and 392 health controls (Table 1). Random-effects meta-analysis demonstrated that patients with PD had significantly lower plasma Zn levels compared with health controls (SMD = −0.73; 95% CI [−1.14, −0.32]; P = 0.000; Fig. 3) with significant heterogeneity among these studies (I^2^ = 77.6%, P = 0.011). We did not perform any further analysis due to the limited number of studies.

### Meta-analysis of Zn levels in the serum and plasma

**Figure 4:**
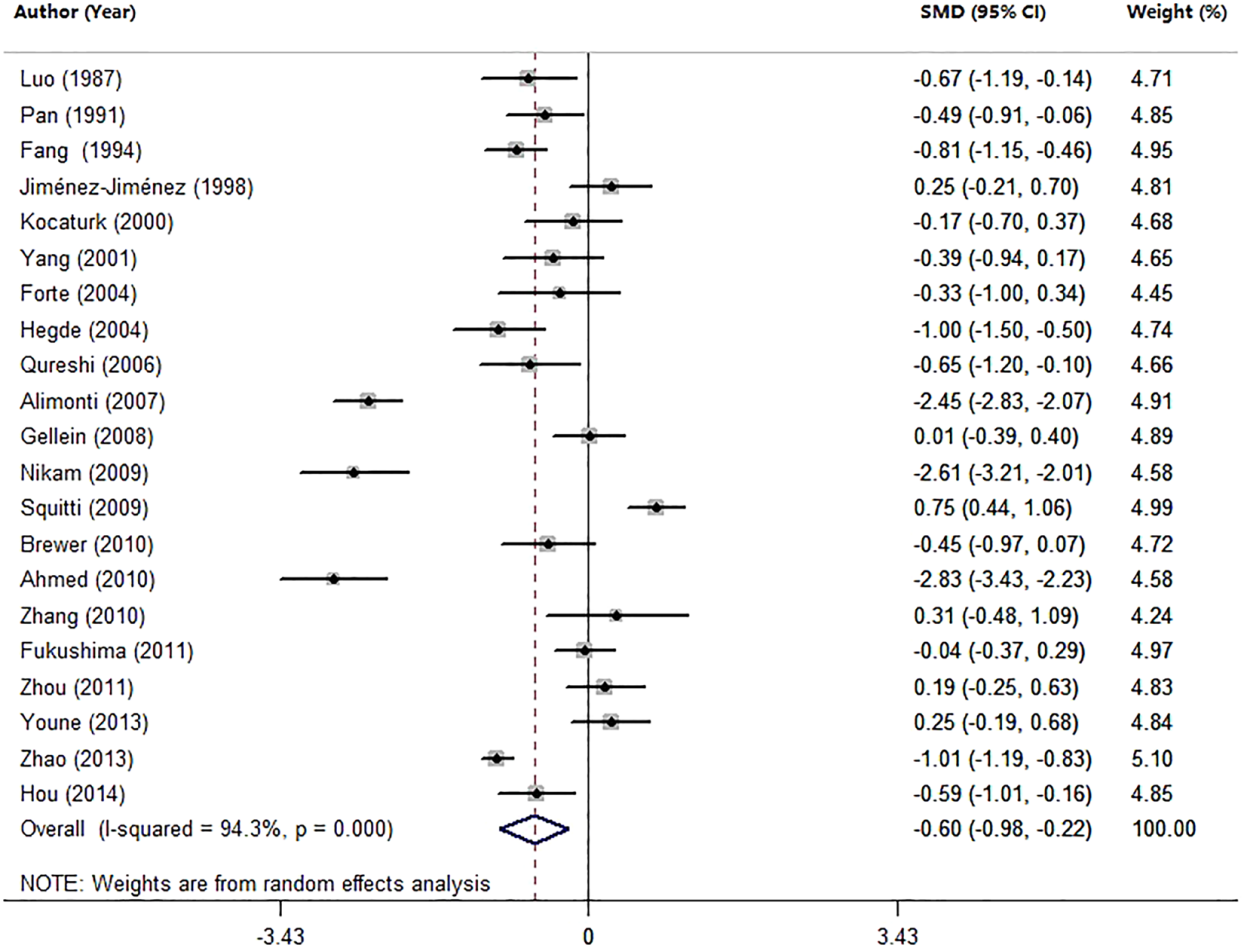
Forest plot for random-effects meta-analysis on differences in serum Zn together with plasma Zn between PD patients and health controls. 21 studies encompassing a sample of 2333 subjects. The horizontal lines represent 95% CI. The sizes of the shaded squares are proportional to study weight. SMD, Standardized mean difference; CI, confidence interval.

We also performed a joint analysis on 21 studies by combining studies investigating zinc levels in the serum and plasma (Table 1). The pooled sample size consisted of 2333 subjects including 1145 PD patients and 1188 heath controls. The Zn levels were significantly decreased in PD patients compared with health controls (SMD = −0.60, 95% CI [−0.98; −0.22]; p = 0.002; Fig. 4) with high heterogeneity (I^2^ = 94.3%, P = 0.000). Subgroup analysis by the method used for measuring Zn levels or by geographic locations of the studied population showed high heterogeneity in each subgroup (Table 3). Meta-regression analyses revealed that mean age and gender had no moderating effects on the outcomes of the meta-analysis (mean age: P = 0.076; gender: P = 0.139). Sensitivity analyses indicated that the results were not unduly changed by a particular study. Temporal effect was not observed by the cumulative meta-analysis. According to the Egger’s test, no evidence of publication bias (P = 0.852) was found.

**Table 3:** Meta-analysis of studies on serum together with plasma.

Subgroups	n of studies	SMD (95% CI)	I^2^	P-value
All studies	21	−0.60 (−0.98, −0.22)	94.3%	0.000
Methods				
ICP-MS or ICP-AES	10	−0.79 (−1.42, −0.16)	94.9%	0.000
AAS	10	−0.56 (−1.01, −0.12)	90.7%	0.000
other	1	0.75 (0.44, 1.06)	—	—
Geographic locations				
Asia	13	−0.77 (−1.17, −0.37)	91.6%	0.000
Europe or America	7	−0.41 (−1.31, 0.49)	96.6%	0.000
Africa	1	0.25 (−0.19, 0.68)	—	—

SMD with the corresponding 95% CI and

p value, the I^2^ statistic for overall and subgroup analyses. SMD, Standardized mean difference; CI, confidence interval.

### Meta-analysis of Zn levels in the CSF

**Figure 5:**
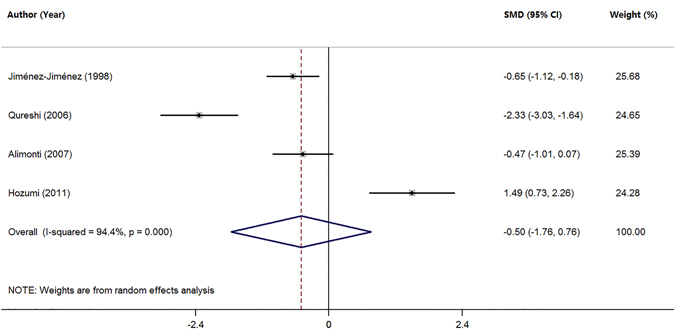
Forest plot for random-effects meta-analysis on differences in CSF Zn between PD patients and health controls. 3 studies encompassing a sample of 229 subjects. The horizontal lines represent 95% CI. The sizes of the shaded squares are proportional to study weight. SMD, Standardized mean difference; CI, confidence interval.

Four studies were in the meta-analysis of Zn levels in the CSF (Table 1). The subject sample consisted of 228 subjects including 135 PD patients and 93 health controls. Random-effects meta-analysis showed that PD patients had a tendency toward reduced Zn levels in the CSF compared with health controls (SMD = −0.50; 95% CI [−1.76, 0.76]; P = 0.439; Fig. 5), but no statistical significance was obtained due to a small sample size. Significant heterogeneity was found among these studies (I^2^ = 94.4%, P = 0.000). As depicted in the forest plot, compared wtih controls, zinc levels in the CSF in PD patients was not significantly different in the Alimonti’s^41^ study, significantly increased in the Hozumi’s^42^ study, and was lower in the Jiménez’s^23^ and Qureshi’s^35^ studies. No further analysis was performed due to the limited number of studies.

## Discussion

It remains unclear whether circulating Zn levels are associated with PD. In this meta-analysis, we found that the Zn levels in the serum and plasma were reduced in PD patients compared with healthy controls. However, strong heterogeneity was found among the studies. Subgroup analyses showed that methods for measuring Zn levels and geographic locations were not the source of the heterogeneity. Furthermore, meta-regression analysis showed that mean age and gender of the patients had no significant effect on the analysis. Despite the high heterogeneity among studies, our study suggests that a reduction in the serum Zn levels is associated with an increased risk of PD. The statistical power was increased when the samples size was increased by pooling of the studies with serum Zn levels and plasma Zn levels. In this meta-analysis, we found that the Zn level in the plasma was significantly lower in PD patients than health controls. However, the small number of studies and high heterogeneity among studies cautions should be taken to interpret the conclusion. In addition, PD patients had a tendency toward reduced CSF Zn levels compared with the health controls. Since the number of studies and the sample size of these studies with CSF Zn levels (4 studies, 135 PD patients and 93 health controls) were relatively small, the association between CSF Zn levels and PD is inconclusive. Further studies with a large sample size are required to investigate the CSF Zn levels in PD.

It is still unclear how Zn reduction affects the progression of PD. It has been reported that Zn can protect against oxidative stress by increasing the superoxide dismutase activities, and inhibiting the nitric oxide synthetase^39, 47^. Thus, Zn deficiency may result in increased oxidative stress, which leads to nigral cell death in PD^26^. This idea is supported by the findings that the levels of nitric oxide and lipid peroxidation products are high and the Zn levels are low in the blood plasma of PD patients^17, 26^. In addition, Zn is required for maintaining the conformation of Parkin, and Parkin dysfunction can cause PD. Since removal of Zn from Parkin can cause unfolding of the protein, Zn deficiency may contribute to the progression of PD by inducing Parkin dysfunction^22^. Interestingly, in the Drosophila disease model with a defective Parkin protein, Zn-supplemented food intake resulted in an extended lifespan and an improvement of motoric abilities^17, 48^. Although no clinical reports have shown the effectiveness of Zn therapy in patients with PD, Zn therapy has been proven to favor improvement and even resolution of neurological damage in patients with Wilson’s disease^49^. Our findings that PD patients had a lower circulating Zn level support the notion that Zn deficiency is a potential risk factor for PD, and Zn-supplemented intervention may be a potential therapy for PD.

There are some limitations in this meta-analysis. First, although we collected the most comprehensive and updated publications, especially those for CSF or plasma Zn levels, were still limited. Further studies with larger sample sizes of PD patients are required to confirm our conclusion. Second, the dietary intake of metal elements has been reported to be related to risk for PD^6, 50^. However, we could not analyze possible associations between dietary intake of Zn and the risk for PD due to limited data of the included studies. In addition to Zn, Fe and Cu are also essential nutrients in food for human health. Mariani *et al*. found that plasma/serum Fe and Cu in PD patients did not differ from that in healthy controls^51^, whereas Jiao *et al*. reported a higher serum Fe levels in PD patients compared to the healthy controls^52^. Therefore, it seems that we could not explain the reasons for the differences in plasma/serum essential metals only based on differences in dietary intake. The mechanisms underlying disbalance of circulating Zn levels in PD patients remain unknown. Dysfunction of Zn transporters, such as divalent metal transporter 1, ZnT3 and LC30A10, has been found in PD^53–57^. Therefore, the low Zn level in PD patients may be the result of dysfunction of Zn transporters. Third, the mean Zn levels varied greatly among studies. The variability suggested problems with the technique for sampling or maybe the methods used. In addition, we repeated the analysis after excluding the studies with the maximum and the minimum Zn concentrations (Pan 1991; Zhang 2010), and further performed the analysis of the difference in serum Zn levels again. However, the results also showed lower levels of Zn in PD patients compared with health controls (SMD = −0.65; 95% CI [−1.17, −0.13]; P = 0.015), suggesting good stability of our meta-analysis. Forth, we have reviewed the English or Chinese studies, but the studies published in other languages were excluded, though these studies might meet the inclusion criteria.

In conclusion, to the best of our knowledge, this meta-analysis is the first study that investigated the association of circulating Zn levels in PD patients compared with health controls. Our meta-analysis indicated that the Zn level in the serum and plasma was significantly lower in PD patients compared with health controls. However, the results need to be interpreted with caution because there is a high level of heterogeneity among studies.
